# Shape decomposition algorithms for laser capture microdissection

**DOI:** 10.1186/s13015-021-00193-6

**Published:** 2021-07-08

**Authors:** Leonie Selbach, Tobias Kowalski, Klaus Gerwert, Maike Buchin, Axel Mosig

**Affiliations:** 1 Department of Computer Science, Faculty of Mathematics, Ruhr University Bochum, Bochum, Germany; 2 Department of Biophysics, Faculty of Biology and Biotechnology, Ruhr University Bochum, Bochum, Germany; 3 Bioinformatics Group, Faculty of Biology and Biotechnology, Ruhr University Bochum, Bochum, Germany; 4 Center for Protein Diagnostics, Ruhr University Bochum, Bochum, Germany

**Keywords:** Laser capture microdissection, Shape decomposition, Skeletonization

## Abstract

**Background:**

In the context of biomarker discovery and molecular characterization of diseases, laser capture microdissection is a highly effective approach to extract disease-specific regions from complex, heterogeneous tissue samples. For the extraction to be successful, these regions have to satisfy certain constraints in size and shape and thus have to be decomposed into feasible fragments.

**Results:**

We model this problem of constrained shape decomposition as the computation of optimal feasible decompositions of simple polygons. We use a skeleton-based approach and present an algorithmic framework that allows the implementation of various feasibility criteria as well as optimization goals. Motivated by our application, we consider different constraints and examine the resulting fragmentations. We evaluate our algorithm on lung tissue samples in comparison to a heuristic decomposition approach. Our method achieved a success rate of over 95% in the microdissection and tissue yield was increased by 10–30%.

**Conclusion:**

We present a novel approach for constrained shape decomposition by demonstrating its advantages for the application in the microdissection of tissue samples. In comparison to the previous decomposition approach, the proposed method considerably increases the amount of successfully dissected tissue.

## Introduction

Laser capture microdissection (LCM) [1] is a highly effective approach to extract specific cell populations from complex, heterogeneous tissue samples. In the dissection, a laser cuts around the boundary of a selected region and a subsequent laser pulse catapults the fragment into a collecting device. LCM has been used extensively in the context of biomarker discovery [2] as well as the molecular characterization of diseases [3]. Since LCM separates homogeneous and disease-specific regions from their heterogeneous and unspecific surrounding tissue regions, the characterizations obtained from genomic, transcriptomic or proteomic characterizations of samples processed with LCM provide more accurate molecular markers of diseases [4, 5]. With LCM being used more and more commonly in clinical studies, there is a need to automate all procedures involved in sample processing.

### Practical application

Our contribution is motivated by an application introduced in [2] in which a region of interest (ROI) to be dissected from the tissue sample is identified using label-free hyperspectral infrared microscopy. In this approach, an infrared microscopic image of the sample yields infrared pixel spectra at a spatial resolution of about 5 μm. A previously trained random forest classifier assigns each pixel spectrum to one tissue component such as *healthy* or *diseased*, with the *diseased* class being further subdivided into *inflamed tissue* as well as several subtypes of thoracal tumors. The general sample preparation task in the context of LCM is to dissect all tumor regions (or all regions identified as one specific tumor subtype) from a sample. The current standard approach for the dissection with LCM is to draw shapes manually. This is severely limited, not only because it takes an inacceptable amount of time for large numbers of samples, but also because it is required that the human operator will subjectively decompose complex regions into smaller fragments. In this paper, we propose a novel automated decomposition approach. While our current contribution deals with the specific context of label-free infrared microscopy, our approach equally applies more broadly to LCM in the context of other microscopic modalities, most notably H&E stained (hematoxylin and eosin stained) images [4] for which recent digital pathology approaches facilitate reliable computational identification of disease specific regions [6, 7].

### Problem statement and solution

In this paper, we address one central problem of processing samples with LCM. That is, not all dissected fragments can be successfully collected due to various possible circumstances. Besides technical reasons as for example an incorrect focus of the laser, the main cause is assumed to be the size and morphology of the fragment. The fragments must not exceed certain limits of minimal or maximal size and should be of approximately round shape. As the regions of interest (ROIs) in tissue samples consist of complex shapes of varying sizes, they oftentimes do not satisfy these constraints and therefore cannot be extracted from the tissue sample without any previous processing. This either increases the amount of necessary user-interaction or negatively affects the sample quality and thus compromises the advantages of LCM-based sample preparation.

**Fig. 1:**
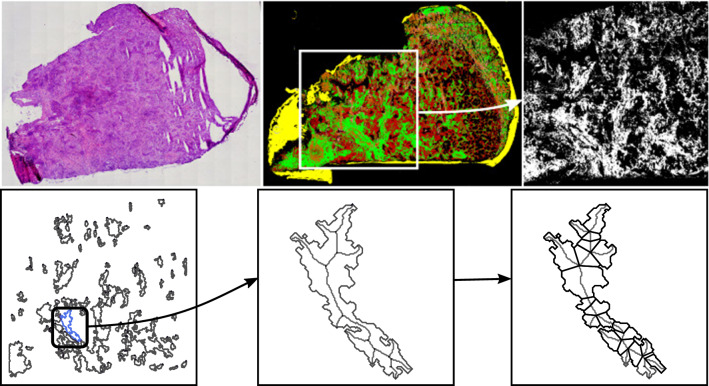
Polygon decomposition in a histopathological tissue sample. Top: Regions of interest are selected from a histopathological tissue sample (H&E-stained image of a subsequent sample on the top left) in which different tissue types have been identified using the method in [2]. Bottom: After a preprocessing, each connected component is given as a simple polygon without holes, which is then decomposed using the proposed skeleton-based approach

Given a binary mask of a microscopic slide with the ROIs as the foreground, the image is preprocessed for LCM in such a way that the ROIs are reduced to a number of connected components without holes. By interpreting each of these connected components as a simple polygon, we can model the given problem of constrained shape decomposition as the computation of optimal feasible decompositions of polygons (see Fig. 1). The constraints can be modeled as certain feasibility criteria and optimization goals. Our decomposition method utilizes a skeleton of the shape and follows a dynamic approach. Specifically, we restrict our cuts to certain line segments based on the skeleton. This not only results in simple cuts but also in a flexible framework that allows to integrate various criteria.

With this paper, we present a novel approach for the automated decomposition of tissue samples with limited user-interaction. Unlike previous decomposition methods used in the context of LCM, we placed a focus on the morphological properties of the fragments. In the experimental evaluation on lung tissue samples of patients with non-small-cell lung carcinoma, the proposed approach achieved a higher success rate and the amount of successfully collected tissue was increased by 10-30%. This paper is an extended version of the preliminary work presented in [8].

The paper is organized as follows: In "Related work" section, we discuss related work on decomposition algorithms. In "Method" section, we introduce our algorithmic framework and discuss different possible feasibility criteria as well as optimization goals. In "Experimental results" section, we present experimental results and demonstrate the advantages of our method in comparison to a heuristic decomposition approach. We conclude with a summary of our results and future improvements in "Conclusion" section.

## Related work

Polygon decomposition is an important tool in computational geometry, as many algorithms work more efficiently on certain polygon classes, for example convex polygons [9]. Moreover, polygon decomposition is frequently used in applications such as pattern recognition or image processing [9]. Object recognition, biomedical image analysis and shape decomposition are typical areas of application that utilize skeletons [10]. Skeletons are oftentimes used to analyze the morphology of a given shape and work especially well on elongated structures, such as vessels [11], pollen tubes [12] or neuron images [13, 14]. There are several shape decomposition methods based on the skeleton or some other medial representation of a shape. However, most of these methods are designed for object recognition and thus focus on decomposing a shape into “natural” or “meaningful” parts [15–17]. In some approaches, even decompositions with overlapping parts are allowed [18, 19]. None of the established decomposition methods facilitate a straightforward introduction of adjustable size and shape constraints as needed for our application.

We utilize the skeleton for two main reasons: it is well-established to represent shape morphology and has proved useful for shape decomposition. As cancerous tissue regions often present themselves as highly complex and ramified shapes, we apply the skeleton to obtain a morphological representation, based on which we compute a decomposition that includes the morphological features.

## Method

To improve the success rate of LCM, a shape decomposition method is needed that computes feasible fragments, i.e. fragments that fulfill certain constraints in size and morphology. We propose an algorithm for constrained polygon decomposition using a skeleton-based approach.

### Skeletonization

Our approach is based on the medial axis or *skeleton* of the shape. The medial axis is defined as the set of points that have more than one closest point on the boundary of the shape. The medial axis was introduced for the description of biological shapes [20, 21] but is now widely used in other applications such as object recognition, medical image analysis and shape decomposition (see [10] for a survey). An important property is that the medial axis represents the object and its geometrical and topological characteristics while having a lower dimension [22, 23].

**Fig. 2:**
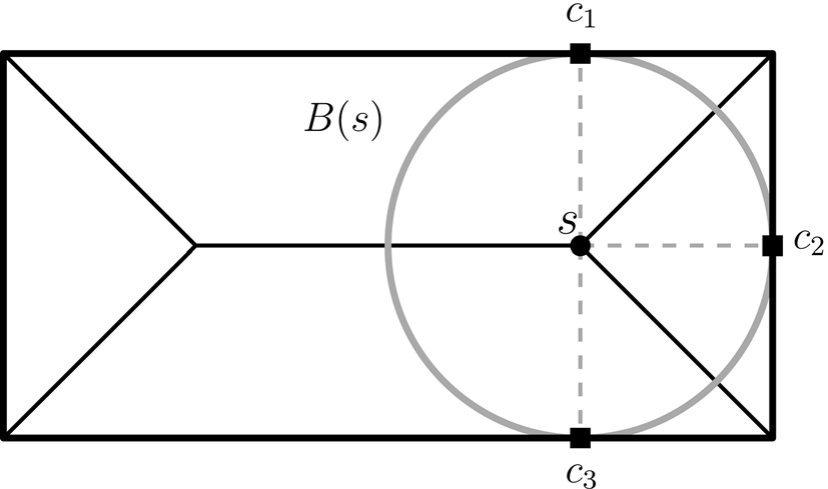
Medial axis of a simple shape. This medial axis consists of five branches connected by two branching points. The skeleton point *s* is the center of a maximal disk *B*(*s*) and has three contact points $\{c_1,c_2,c_3\}$ on the boundary of the shape

Formally, the medial axis of a shape *D* is defined as the set of centers of maximal disks in *D*. A closed disk $B\subset D$ is maximal in *D* if every other disk that contains *B* is not contained in *D*. A point *s* is called *skeleton point* if it is the center of a maximal disk *B*(*s*) (see Fig. 2). For a skeleton point *s*, we call the points where *B*(*s*) touches the boundary the *contact points*—every skeleton point has at least two contact points. A skeleton *S* is given as a graph consisting of connected arcs $S_k$, which are called *skeleton branches* and meet at *branching points*. Given a simple polygon without holes the skeleton is an acyclic graph.

There are various methods for the computation of the medial axis in practice [10]. In general, the medial axis is very sensitive to noise in the boundary of object. This is a problem that often occurs in digital images and leads to spurious skeleton branches. Procedures that remove these uninformative branches are known as pruning methods. Pruning can be applied after skeletonization [24–26] or is included in the computation of the skeleton [27–30]. For our application, we utilize the skeletonization and pruning method of Bai et al. [29], which was previously used for other bioimaging applications [12, 14]. This algorithm produces a discrete and pruned skeleton, which consists of a finite number of skeleton pixels as our skeleton points. This is favorable for our practical application as we have a discrete input and a discrete output is expected. Furthermore, the computed skeleton has the property that every branching point has a degree of exactly three.

### Skeleton-based polygon decomposition

We consider the following problem: Given a simple polygon *P*, compute an optimal feasible decomposition of *P*. A decomposition is *feasible* if every subpolygon is feasible, in the sense that it fulfills certain conditions on for instance its size and shape. We present an algorithmic framework that allows the integration of various criteria for both feasibility and optimization, which are discussed later. As for now, we only consider criteria that are locally evaluable.

In our skeleton-based approach, we only allow cuts that are line segments between a skeleton point and its corresponding contact points. Thus, the complexity of our algorithm mainly depends on the number of skeleton points rather than the number of boundary points of the polygon. Every subpolygon in our decomposition is generated by two or more skeleton points. We present two decomposition algorithms: One in which we restrict the subpolygons to be generated by exactly two skeleton points and a general method. In the first case, each subpolygon belonging to a skeleton branch can be decomposed on its own and in the second case the whole polygon is decomposed at once.

#### Decomposition based on linear skeletons

**Fig. 3:**
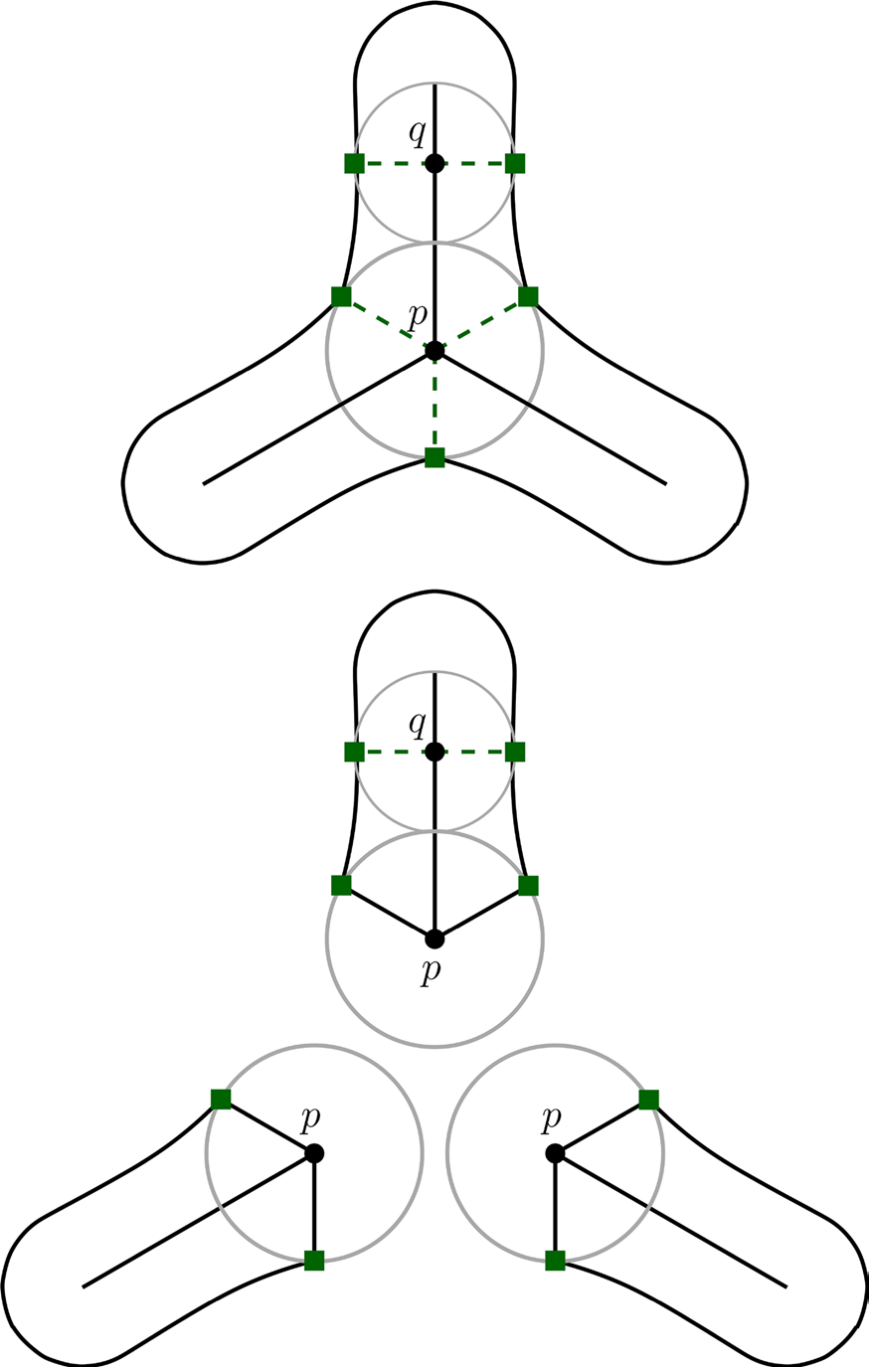
Domain decomposition lemma. The domain is decomposed based on the contact points of skeleton point *p*. The partial skeletons share only *p* as a common point. All contact points of any other skeleton point *q* are contained in exactly one of the connected components

First, we consider the restriction that the subpolygons are generated by exactly two skeleton points. In this case, the corresponding skeleton points have to be on the same skeleton branch $S_k$. In our computed skeleton, a branching point belongs to exactly three branches and thus has three contact points. Each combination of two out of the three possible cut line segments corresponds to one of these branches. Due to the Domain Decomposition Lemma (see Fig. 3, proof in [22]) and the following corollary, we can decompose each skeleton branch on its own.

##### **Theorem 1**

(Domain Decomposition Lemma) *Given a domain* *D* *with skeleton* *S*(*D*), *let* $p\in S(D)$ *be some skeleton point and let* *B*(*p*) *be the corresponding maximal disk. Suppose* $A_1,A_2,\ldots ,A_k$ *are the connected components of* $D\setminus B(p)$. *Define* $D_i = A_i \cup B(p)$ *for all* *i*. *Then:*

$$\begin{aligned} S(D) = \bigcup _{i=1}^k S(D_i). \end{aligned}$$

*Moreover, we have*

$$\begin{aligned} S(D_i) \cap S(D_j) = {p} \;\forall \; i\ne j. \end{aligned}$$

##### **Corollary 2**

*Let* $p\in S(D)$ *and* $A_1,A_2,\ldots ,A_k$ *be as above. For each skeleton point* $q\ne p$ *exists an* *i* *such that all contact points of* *q* *are contained in* $A_i$.

**Fig. 4:**
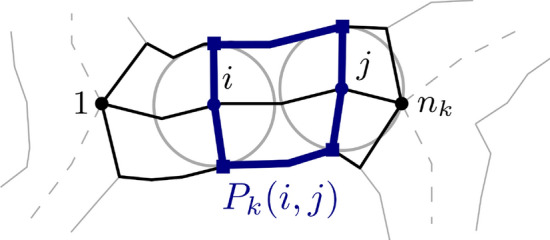
Subpolygon induced by skeleton points. The polygon $P_k$ belongs to the skeleton branch $S_k$ consisting of the points 1 to $n_k$. Two skeleton points *i* and *j* together with line segments to their corresponding contact points induce a subpolygon $P_k(i,j)$

Let $S_k$ be a skeleton branch with a linear skeleton of size $n_k$ and let $P_k$ be the polygon belonging to this branch. By $P_k(i,j)$, we denote a subpolygon that is generated by two skeleton points *i* and *j* on $S_k$ (see Fig. 4). Thus, we have $P_k(1,n_k)=P_k$. First, we consider the decision problem, which can be solved by using dynamic programming. For each skeleton point *i* from $n_k$ to 1, we determine *X*(*i*). *X*(*i*) is True if there exists a feasible decomposition of the polygon $P_k(i,n_k)$. This is the case if either

- $P_k(i,n_k)$ is feasible or
- there exists $j>i$ such that $P_k(i,j)$ is feasible and $P_k(j,n_k)$ has a feasible decomposition.

**Fig. 5:**
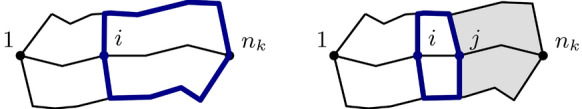
Decomposition based on linear skeletons. The polygon $P_k(i,n_k)$ has a feasible decomposition if either polygon itself is feasible (left) or there exists a point *j* such that $P_k(i,j)$ is feasible and $P_k(j,n_k)$ has a feasible decomposition (right)

This is illustrated in Fig. 5. By choosing optimal points *j* during the computation, we can include different optimization goals. If *X*(1) is True, the entire polygon has a feasible decomposition, which can be computed via backtracking.

##### **Lemma 3**

*Given a subpolygon* $P_k$ *with a linear skeleton* $S_k$ *consisting of* $n_k$ *points, one can compute a feasible decomposition of* $P_k$ *based on* $S_k$ *in time* ${\mathcal {O}}({n_k}^2F)$, *with* *F* *being a factor depending on the feasibility criteria.*

##### *Proof*

We initialize $X(n_k)=\texttt {True}$. For every skeleton point *i*, for $i=n_k-1$ down to 1, we compute *X*(*i*) such that *X*(*i*) equals True if there exists a feasible decomposition of $P_k(i,n_k)$. To compute *X*(*i*), we consider ${\mathcal {O}}(n_k)$ other values *X*(*j*) for $i<j\le n_k$ and check in time ${\mathcal {O}}(F)$ if the polygon $P_k(i,j)$ is feasible. The correctness follows inductively. $\square$

The factor *F* is determined by the runtime it takes to decide whether a subpolygon is feasible. This factor depends on for instance the number of points in the skeleton or in the boundary of the polygon. We discuss examples in the following "Feasibility constraints and optimization" section. After computing decompositions for each subpolygon corresponding to a skeleton branch, we can combine those to obtain a decomposition of the entire polygon. This leads to the following result.

##### **Theorem 4**

*Given a simple polygon* *P* *with skeleton* *S* *consisting of* *n* *points, one can compute a feasible decomposition of* *P* *based on the skeleton branches of* *S* *in time* ${\mathcal {O}}(n^2F)$, *with* *F* *being a factor depending on the feasibility criteria.*

Note that there might not exist a feasible decomposition of the entire polygon or for certain subpolygons. By using this method, we are able to obtain partial decompositions. Thus, this approach can be favorable in practice.

#### General decomposition

**Fig. 6:**
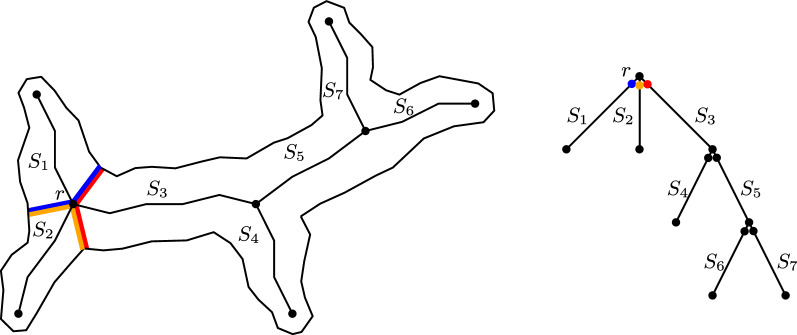
Tree representation of the skeleton graph. Representing the skeleton graph as a tree rooted at the point *r*. Every node in the tree represents a possible cut in the polygon. Therefore the branching points are duplicated to provide the cuts on the respective branches

In the general setting, subpolygons are allowed to be generated by more than two skeleton points. In this paper, we will briefly explain the idea of our method (see [31] for a more detailed description and the corresponding formulas). Recall that our skeleton is an acyclic graph consisting of a finite number of vertices, i.e. skeleton points. The skeleton computed for our application (method of Bai et al. [29]) has the property that the maximal degree of a skeleton point is three. We represent the skeleton as a rooted tree by selecting one branching point as the root (see Fig. 6). Since branching points belong to three different branches, these nodes are duplicated in the skeleton tree such that each node corresponds to the cut edges on the respective branch. Our method and its runtime are based on two main observations.

##### **Observation 5**

The maximal number of skeleton points that can generate a subpolygon is equal to the number of endpoints in the skeleton, i.e. the number of leaves in the skeleton tree.

##### **Observation 6**

Every subpolygon can be represented as the union of subpolygons generated by just two skeleton points.

**Fig. 7:**
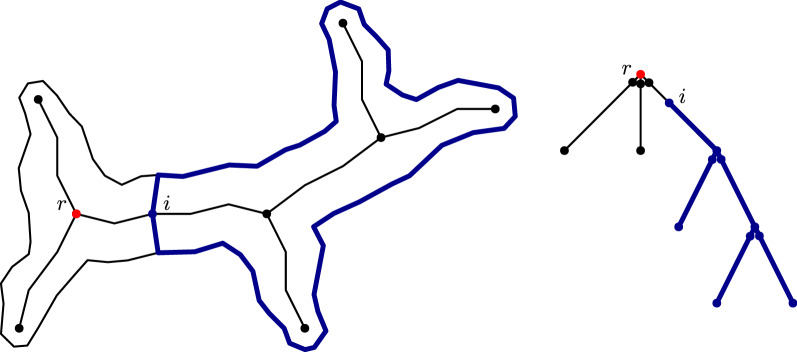
A subpolygon and its corresponding subtree. The subpolygon *P*(*i*) ending in the skeleton point *i* is represented by the subtree $T_i$

Let *i* be a node in the skeleton tree and $T_i$ the subtree rooted in *i*. By *P*(*i*), we denote the subpolygon ending in the skeleton point *i*. This polygon corresponds to the subtree $T_i$ in the given tree representation (see Fig. 7). For each node *i* (bottom-up), we compute if there exists a feasible decomposition of the polygon *P*(*i*). Such a decomposition exists if either

- *P*(*i*) is feasible or
- There exists a feasible polygon $P'$ ending in *i* and feasible decompositions of the connected components of $P(i)\setminus P'$.

**Fig. 8:**
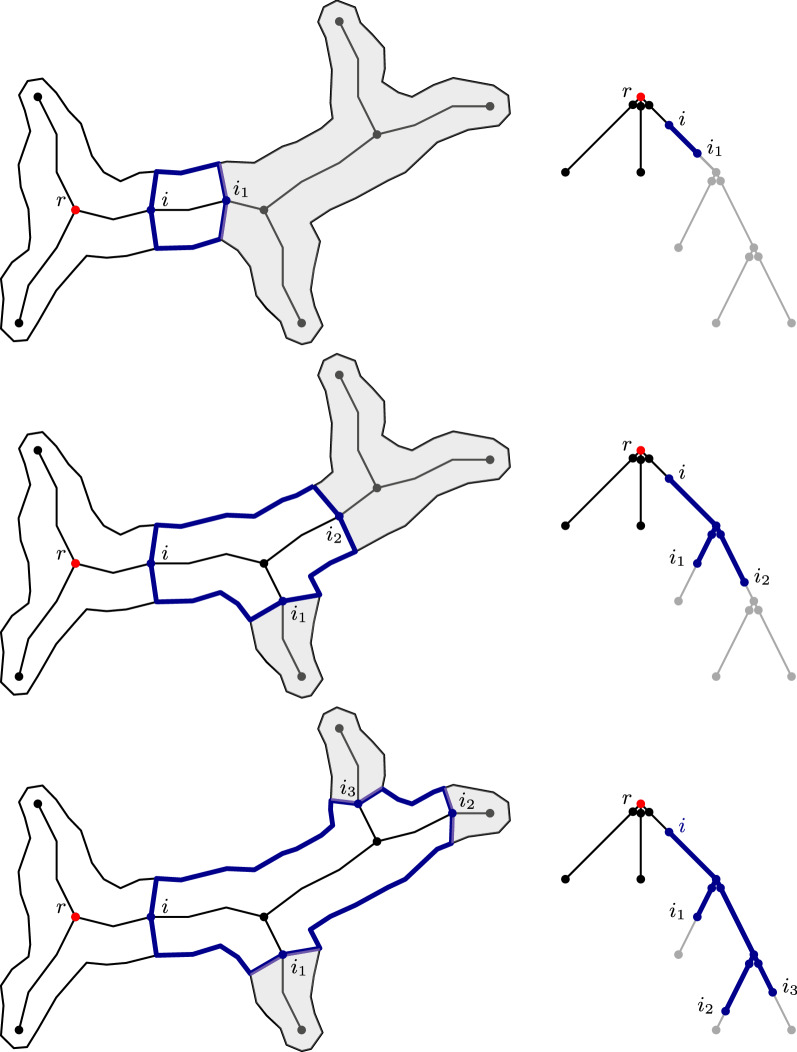
Possible combination of skeleton points considered in the decomposition of a subpolygon. In the decomposition of a subpolygon *P*(*i*), different combinations of skeleton points in the subtree $T_i$ are considered. The resulting subpolygons can be represented as subtrees (blue) in the tree representation spanning between nodes of these skeleton points

Thus, we have to consider all different combinations of skeleton points that together with *i* can form such a polygon $P'$. In a top-down manner, we consider the different combinations of nodes $\left[ i_1,i_2,\ldots ,i_l\right]$ such that $i_j\in T_i$ and $T_{i_j} \cap T_{i_{j'}}= \emptyset$ for all $j\ne j'$. The polygon $P'$ corresponds to the subtree rooted in *i* with $i_1,i_2,\ldots , i_l$ as the leaves, depicted in blue in Fig. 8. Note that we can compute $P'$ as a union of subpolygons iteratively. We check if $P'$ is feasible and if we have feasible decompositions for each $P(i_j)$, meaning every subtree $T_{i_j}$ (gray in Fig. 8). Because of Observation 5, we know that $l\le k$, for *k* being the number of leaves in the skeleton tree. We have a feasible decomposition of the whole polygon if there exists one of the polygon *P*(*r*). This computation dominates the runtime with the maximum number of combinations to consider being in ${\mathcal {O}}(n^k)$. Note that this approach does not depend on the initial choice of the root node.

##### **Theorem 7**

*Given a simple polygon* *P* *with skeleton* *S* *consisting of* *n* *points with degree at most three, one can compute a feasible decomposition of* *P* *based on* *S* *in* ${\mathcal {O}}(n^kF)$ *time, with* *k* *being the number of leaves in the skeleton tree and* *F* *as above.*

### Feasibility constraints and optimization

The proposed polygon decomposition method is a versatile framework that can be adjusted for different feasibility constraints and optimization goals. With regard to the application in LCM, we considered criteria based on size and shape. As stated before, it is assumed that the main cause for unsuccessful dissections lies in an incorrect size or morphology of the considered fragments. In LCM, a laser separates a tissue fragment from its surrounding sample leaving a small connecting bridge as the impact point of a following laser pulse, which catapults the fragment into a collecting device. As the laser burns part of the boundary, the fragment has to have a certain minimal size to ensure that enough material is supplied to be analyzed. On the other hand, the size cannot be too large or otherwise the force of the laser pulse does not suffice for the transfer process. Furthermore, observations show that the dissection often fails due to an irregular shape of the fragment. Specifically, elongated shapes or fragments with narrow regions (bottlenecks) seem to be problematic. The tissue can tear at these bottlenecks and is only transferred partly or not at all because the laser pulse is concentrated on only a small part of the boundary.

In the following, we describe the implementation of different constraints that we considered based on our application. For simplicity, we limit the description to the decomposition algorithm for linear polygons, but all mentioned constraints can be applied to the general decomposition method as well.

#### Feasibility constraints

For the size constraint, we restricted the *area* of the subpolygons. Given two bounds *l* and *u*, a polygon *P* is feasible if $l\le A(P)\le u$, for *A*(*P*) being the area of the polygon. One could also apply this constraint on the number of boundary points instead of the area. We implemented different shape constraints. On the one hand, we considered *approximate convexity*. Then, a polygon *P* is feasible if every inner angle lies between two given bounds. As this criterion does not prevent elongated shapes, we considered *fatness* instead. Fatness can be used as a roundness measurement and is defined by the aspect ratio *AR*(*P*) of a polygon, which is the ratio between its width and its diameter [32–34]. For a simple polygon, the diameter is defined as the diameter of the minimum circumscribed circle and the width as the diameter of the maximum inscribed circle. A polygon *P* is called $\alpha$-fat if $AR(P)\ge \alpha$. For the fatness constraint, we define a polygon as feasible if it is $\alpha$-fat for some given parameter $\alpha \in \left( 0,1\right]$. Higher values of $\alpha$ result in fragments that are more circular and less elongated in shape.

For area as well as approximate convexity, we can compute the required values incrementally if the values for all subsequent subpolygons are given beforehand. Therefore, we can check the feasibility in constant time. Thus, a feasible decomposition using these criteria can be computed in time ${\mathcal {O}}(n^2+m)$ for *n* being the number of skeleton points and *m* the number of boundary vertices. If the fatness criterion is used, one has to calculate the aspect ratio of each polygon, which takes ${\mathcal {O}}(m\log m)$ time and therefore results in a runtime of ${\mathcal {O}}(n^2m\log m)$.

#### Optimization goals

The algorithm computes the value *X*(*i*) for each skeleton *i*. For the decision problem, we defined *X*(*i*) to be True if there exists a feasible decomposition of the polygon *P*(*i*, *n*). With a redefinition of *X*(*i*), we can implement a variety of optimization goals. For a point *i*, let *I* be the set of points *j* such that *P*(*i*, *j*) are feasible. One possible optimization goal is finding a minimal decomposition by *minimizing the number of fragments* (MinNum). We define *X*(*i*) as the number of subpolygons in an optimal feasible decomposition of *P*(*i*, *n*), set $X(n) = 0$ and compute $X(i) = \min _{j\in I}X(j)+1$. By *minimizing the length of cut edges* (MinCut), shorter cuts are preferred and thus preferably placed at bottlenecks in the polygon. Since every skeleton point *i* is the center of a maximal disk, we can obtain the cut length by the corresponding radius *r*(*i*). We can define *X*(*i*) either as the length of the longest cut or as the sum of cut lengths in an optimal decomposition of *P*(*i*, *n*) and compute $X(i) = \max \{\min _{j\in I}X(j), r(i)\}$ or $X(i)=\min _{j\in I}X(j)+r(i)$. The runtime for both MinNum and MinCut is the same as for the decision problem. Furthermore, we considered *maximizing the fatness* (MaxFat) as an optimization goal. A decomposition is optimal if the smallest aspect ratio is maximized. We define *x*(*i*) as the value of the smallest aspect ratio and compute $X(i) = \max _{j\in I}\{\min \{X(j),AR(P(i,j))\}\}$. Applying fatness as a feasibility constraint or an optimization goal results in the same runtime because both approaches require the calculation of the aspect ratios of all subpolygons.

#### Comparison of criteria

Our algorithm facilitates the use of a wide range of feasibility criteria and optimization goals, which can be combined with each other. Note that for certain combinations other (faster) methods might exist. One example is finding the minimal (MinNum) decomposition in which the area of the subpolygons is bounded. For polygons with linear skeletons this can be modeled as finding the minimal segmentation of a weighted trajectory (in ${\mathcal {O}}(n\log n)$ time [35]). For general polygons, this problem can be modeled as computing the minimal (*l*, *u*)-partition of a weighted cactus graph (in ${\mathcal {O}}(n^6)$ time [36, 37]).

**Fig. 9:**
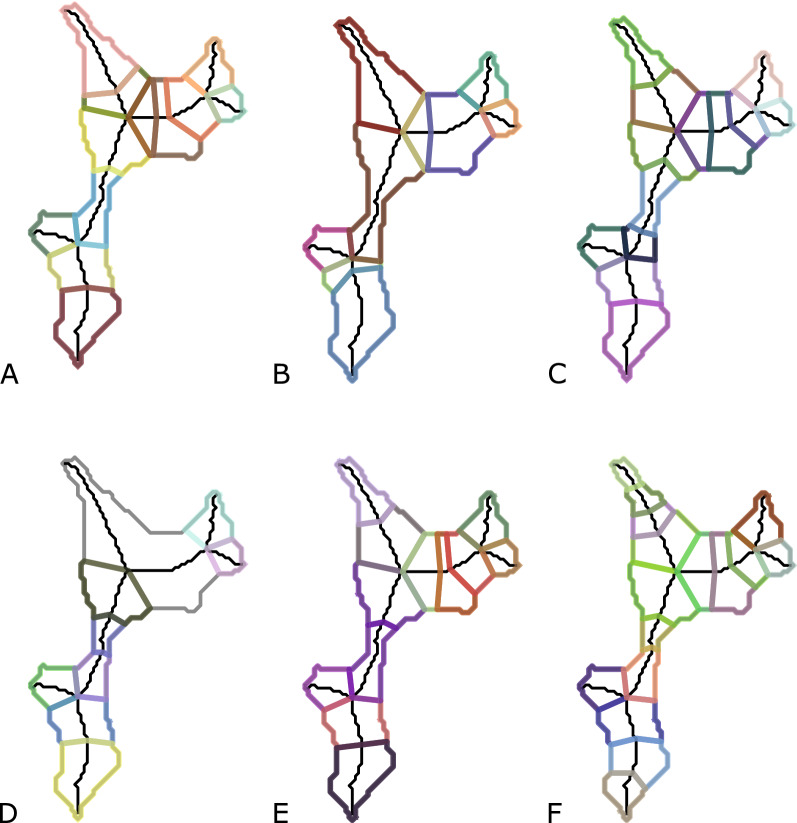
Decompositions based on different criteria. Exemplary decompositions obtained by applying the algorithm based on linear skeletons with different feasibility constraints and optimization goals. **A** area in $\left[ 50,300\right]$, MinNum. **B** area in $\left[ 50,500\right]$, MinNum. **C** area in $\left[ 50,300\right]$, fatness $\ge$ 0.4, MinNum. **D**area in $\left[ 50,300\right]$, fatness $\ge$ 0.5, MinNum. **E** area in $\left[ 50,300\right]$, MinCut. **F** area in $\left[ 50,300\right]$, MaxFat

The selection of constraints used for the algorithm obviously affects the resulting decomposition. The number of subpolygons as well as the position of cuts varies noticeably. Depending on the underlying application, one might choose suitable constraints. In the following, we present decompositions for different combinations of criteria and assess their suitability for our specific application. The typical results are exemplified using a ROI polygon of a lung tissue sample (see Fig. 9).

Panel A and B in Fig. 9 illustrate the effect of the size criterion. Having a larger upper bound obviously results in fewer subpolygons. The MinNum optimization goal minimizes the number of subpolygons, but the solutions are not necessarily unique and one optimal decomposition is chosen arbitrarily. This can be observed at the bottom-most skeleton branch in both these decompositions as the cuts in A would be feasible with the constraints from B as well. In panel C and D of Fig. 9, the fatness constraint was applied in form of a lower bound on the aspect ratio of subpolygons. In comparison to the decomposition depicted in A, this criterion avoids the tendency towards elongated fragments. However, tighter bounds do not necessarily result in better outcomes as a feasible decomposition might not exist at all. This case is illustrated in panel D, where the algorithm did not find a feasible decomposition for the polygon parts that are depicted in gray. For our application, this would not be favorable as it reduces the amount of extracted tissue material.

We applied the different optimization goals denoted by MinNum (panel A), MinCut (panel E) and MaxFat (panel F) with the same feasibility constraint. Choosing a different optimization goal will not influence the amount of area in the decomposition, but the quantity and positions of cut edges may change considerably. These changes are expected to affect the amount of successfully dissected tissue fragments in the microdissection. When looking at the decompositions of the top left skeleton branch in those three polygons, one notices that the ones in A and E have the same number of fragments, but with MinCut a cut with a lower length is chosen. Maximizing the fatness usually results in a higher number of subpolygons. As can be seen in panel F, the resulting subpolygons are less elongated and more circular in shape. We expect these to be the desired shapes for our application. Hence, we used the area constraint in combination with the MaxFat optimization in our experiments and the following comparison of decomposition methods for LCM.

## Experimental results

### Experimental setup

For the evaluation of our algorithms, we conducted LCM experiments on shapes obtained from infrared microscopic images of 10 thin sections of FFPE (formalin-fixed paraffin-embedded) lung tissue samples from patients with non-small-cell lung carcinoma. The pixel spectra of the images were classified into different tissue types using a random forest classifier as described in [2]. All pixel positions belonging to the tumor class were chosen as the regions of interest (ROI). The binary mask of the ROI was preprocessed by a morphological opening followed by a morphological closing and subsequent hole filling. Each connected component of the preprocessed binary mask is given as a simple polygon on which we applied two different decomposition approaches. The number of input polygons for our experiments ranged from 14 to 109 per sample with a total amount of 441. The resulting decompositions serve as the input for LCM. Each fragment is transmitted in form of a circular list of discrete boundary points.

**Fig. 10:**
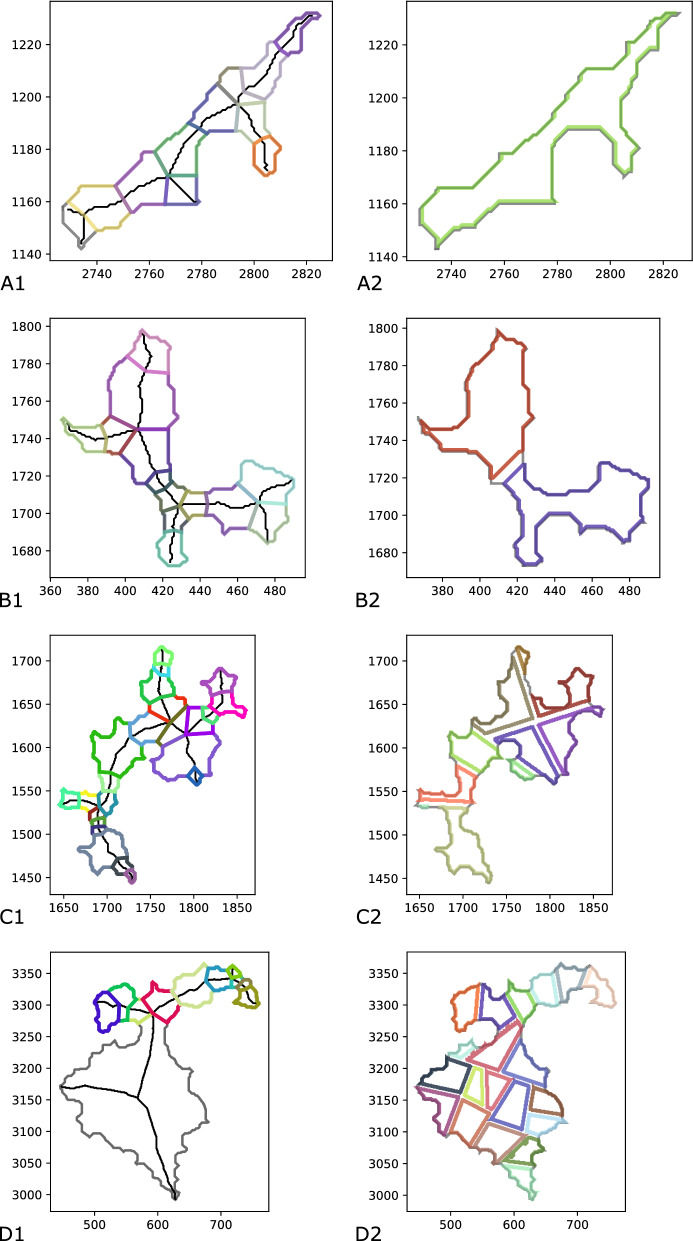
Exemplary decompositions with MaxFat and BiSect. Decompositions of four exemplary components from the tissue samples. Left: decomposition with MaxFat. Right: decomposition with BiSect

For the experiment, we used our algorithm based on linear skeletons such that each skeleton branch is decomposed separately. This follows the practical consideration that a polygon as a whole may not possess a feasible decomposition, while some individual branches do. The resulting skeletons consisted of roughly 80 to 1000 points, involving around five to ten branches for each polygon to be decomposed, see Figs. 9, 10 for typical examples. We applied a size constraint as lower and upper bounds on the area of the subpolygons and computed an optimal decomposition in which the fatness, i.e. the minimum aspect ratio of the subpolygons, is maximized. We denote this approach by *MaxFat*.

We compare our approach to a heuristic decomposition method, which was used to decompose tissue samples for LCM in previous work. As this method follows a bisection approach, we denote it by *BiSect*. Unlike MaxFat, this method includes merely a size constraint and no shape criterion or optimization goal. A polygon is decomposed by recursive bisection if its area exceeds an upper size bound. If the area of a (sub)polygon is below a given lower bound, it is discarded. Every bisection is designed to leave a strip of tissue behind such that each subpolygon retains contact to the surrounding membrane of the microscopic slide in order to meet a technical requirement of the specific LCM system used in this study for the dissection to be possible. The MaxFat decomposition does not include these strips because all subpolygons intersect with the boundary of the input polygon.

Both decomposition methods were applied with the same area bounds, namely a minimal and maximal area of 100 px (ca. 1800 μm^2^) and 2800 px (ca. 50000 μm^2^) respectively. The main focus of the development of a novel decomposition method lies in the reduction of tissue loss. This is determined by the amount of area in the decomposition itself as well as the amount of successfully dissected fragments. First, we compare the methods and the resulting decompositions on a computational level. Then, we analyze their performance in the practical setting with LCM.

### Computational results

We examine the results of the MaxFat and BiSect decomposition on three different levels: fragments (subpolygons), components (ROI polygons) and samples. We examine the size of the decompositions, the area loss and the morphology of the fragments.

#### Decomposition size

The sampling consisted of 441 components with an average area of 4500 px (ca. 81300 μm^2^). The MaxFat decomposition over all ten samples contained 4143 fragments with an average of 9.36 fragments per component. The BiSect decomposition consisted of considerably less fragments with an average of 2.36 fragments per component and 1089 for the entire sampling.

BiSect achieves a smaller decomposition size because most components did not require many bisections for the fragments to fulfill the given area constraints (see Fig. 10 A2, B2). With MaxFat, every skeleton branch in decomposed individually. Therefore, most decompositions consist of at least as many fragments as there are branches in the skeleton (see Fig. 10 A1, B1).

#### Area loss

**Fig. 11:**
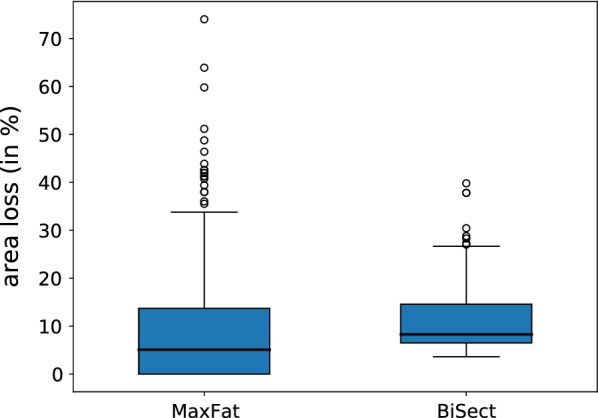
Comparison of area loss for components. Distribution of the area loss (in%) in the decompositions of individual components contained in the sampling (n = 441). Comparison of MaxFat (M = 8.96%, SD = 11.26) and BiSect (M = 10.81%, SD=6.11)

Figure 11 depicts the area loss on the level of individual components. The mean area loss with MaxFat is slightly lower than the one with BiSect (MaxFat M = 8.96%, BiSect M = 10.81%). However, the distribution of MaxFat shows a greater variability in values, a larger standard deviation and some high-loss outliers (MaxFat SD = 11.26, BiSect SD = 6.11). For BiSect, the variability is lower and there are less outliers. On the level of samples, one can see that in 8 of 10 cases the area loss with MaxFat is lower than the one with BiSect (see Table 1). The decompositions with MaxFat contained up to 10% more area. The area loss averages around 10.77% for MaxFat and 16.35% for BiSect.

Both methods inherently involve area loss. With BiSect, area loss occurs due to the strips left behind by every bisection. Therefore, the amount increases proportional to the size of the components and the necessary cuts (see Fig. 10). With MaxFat, area is lost for each skeleton branch for which a feasible decomposition did not exist. This mainly occurs if the corresponding (sub)polygon is either too slim or too wide. The first case is depicted in panel A1 of Fig. 10: Because the area of the gray polygons belonging to the bottom two branches was below the given lower bound, a feasible decomposition did not exist and their area was lost. This can be attributed to shortcomings of the underlying skeleton pruning method. Improving the pruning of the skeleton may avoid such short branches. The second case of too wide shapes is exemplified in panel D1. If the upper area bound is relatively small, the MaxFat decomposition of a wide shape leads to either thin-slicing or no feasible solution at all. This is due to our definition of the cut edges, which do not allow internal decompositions. This also illustrates that our approach is tailored towards more complex, ramified shapes rather than fat objects. It is noteworthy that the polygon depicted in panel D1/D2 of Fig. 10 covers an area of around 43,000 px (ca. 7,77,000 μm^2^) and thus represents a huge outlier in our sampling.

These observations coincide with the presented results. For MaxFat, the first cause of area loss might occur frequently but merely contributes a small value to the overall loss. The second cause does not appear as often in the samples because the average area of the components is fairly small, but obviously results in a large amount of area loss. This contributes to the higher standard deviation and outliers that were observable on the level of individual components. The results for entire samples suggest that the samples are dominated by components that cause small or no area loss when decomposed with MaxFat. Since the resulting fragments for each component in one tissue sample are collectively gathered, the quality of the decomposition should be assessed on the level of samples. Regarding area loss during decomposition, MaxFat generally achieved better results. However, the quality of the methods is ultimately determined by their performance in practice and their success with LCM. Therefore, practical evaluations are necessary. Here, we expect the morphology of the fragments to be a critical factor.

**Table 1:** Comparison of area loss for samples as the combined loss over all components for MaxFat and BiSect

Sample	1	2	3	4	5	6	7	8	9	10
MaxFat	8.58	21.43	5.22	23.85	3.42	11.71	3.40	13.56	10.45	6.07
BiSect	10.62	18.04	13.44	23.41	13.47	19.02	13.00	14.59	18.48	19.46

#### Morphology

**Fig. 12:**
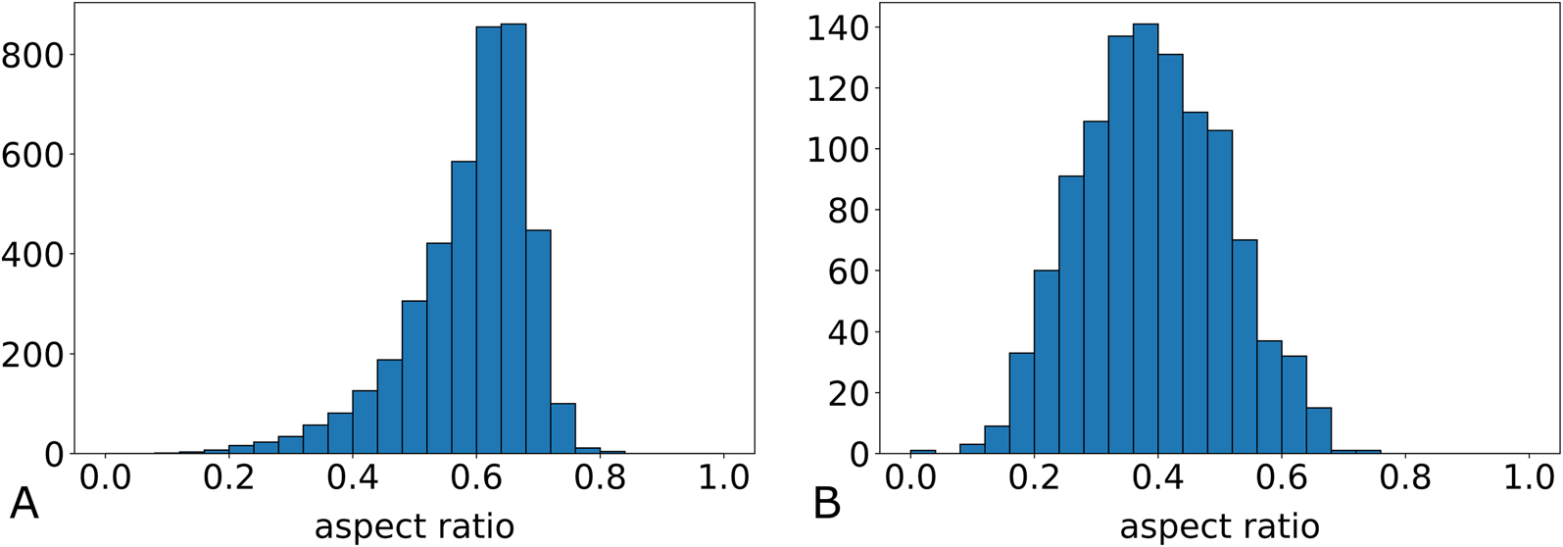
Comparison of the fatness for fragments. Distribution of aspect ratios of individual fragments contained in the decompositions with MaxFat in A (n = 4151, M = 0.58, SD = 0.1) and BiSect in B (n = 1089, M = 0.39, SD = 0.11)

**Fig. 13:**
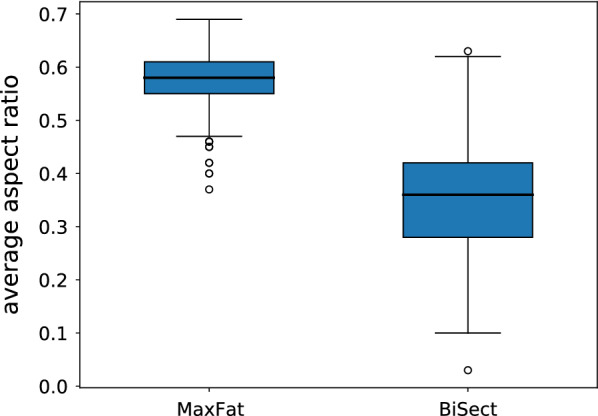
Comparison of the average fatness for components. Distribution of average aspect ratios in the decompositions of all individual components contained in the sampling (n = 441). Comparison of MaxFat (M = 0.58, SD = 0.05) and BiSect (M = 0.36, SD = 0.1)

We compared both decomposition methods based on the resulting fatness, i.e. aspect ratio, of the fragments. This value measures the circularity of the shape. On the level of individual fragments, the aspect ratios in BiSect present themselves in the pattern of a normal distribution whereas the distribution for MaxFat is clearly left-skewed (see Fig. 12). The average aspect ratio of fragments over all samples is considerably higher with MaxFat (MaxFat M = 0.58, BiSect M = 0.39). We observe similar results when considering the average aspect ratio in the decompositions of components (see Fig. 13). The values for MaxFat are larger and the variability is smaller (MaxFat M = 0.58, BiSect M = 0.36). The standard deviation for MaxFat is half as high as the one of BiSect (MaxFat SD = 0.05, BiSect SD = 0.1). For over 75% of components, the average fatness in the decompositions computed with MaxFat was higher than 0.5, whereas with BiSect nearly 75% have an average fatness lower than 0.4.

BiSect applies only a size constraint and a component is only decomposed if its area exceeds the given upper bound. Therefore, many components are not decomposed, but their shape is oftentimes elongated and ramified as can be seen in panel A2 of Fig. 10. Because this method follows a bisection approach, the cut placement creates fragments with irregular shapes and narrow bottlenecks (see Fig. 10 C2). The exception can be observed in large, round components as their decomposition resembles a grid pattern (see panel D2 of Fig. 10). In this case, the resulting fragments achieve a higher fatness. The results suggest that without the application of some shape criterion the BiSect decomposition does not naturally result in fragments of large fatness. MaxFat, on the other hand, utilizes both size and shape criteria and tries to maximize the fatness of a decomposition. This results in smaller fragments that are less elongated and rounder in shape (see Fig. 10). The computational evaluation reveals that MaxFat consistently obtains higher fatness values. This strengthens our choice to include the fatness criterion in the optimization goal rather than the feasibility constraints. Even without applying a strict bound on the fatness, we were able to achieve high fatness values without the risk of area loss due to the non-existence of a feasible decomposition. The success of a dissection using LCM depends on the size and morphology of the tissue fragment. We hypothesize that approximately round shapes have a higher chance to be successfully collected. As MaxFat consistently obtains such fragments, we suspect this to be the main advantage of this decomposition method in the practice. Hence, we expect a higher success rate in the practical application with LCM.

#### Running times

The computations were executed on a Windows PC (Intel Core i5-8600 CPU, 16 GB RAM). The proposed approach consists of a skeletonization and a subsequent decomposition with MaxFat. The average computation time for one input polygon was 0.63 s for the skeletonization, 14.38 s for the decomposition with MaxFat and 0.43 s for the decomposition with BiSect. When considering median values, we see that one for MaxFat (0.24 s) is lower than the one for BiSect (0.47 s). This suggests that MaxFat can perform very fast on the majority of inputs but more slowly on others. In general, the running time for BiSect fairly low, as many polygons are not decomposed at all. For MaxFat, on the other hand, the time complexity depends on the number of boundary points as well as the number of skeleton points.

We looked at one sample in more detail. Sample 7 consisted of 63 polygons with different boundary (M = 274.63, Min = 131, Max = 1104) and skeleton (M = 152.55, Min = 81, Max = 507) sizes. Therefore, the MaxFat decomposition showed a variation in runtimes (M = 1.77 s, Min = 0.21 s, Max = 29.57 s). The runtimes for BiSect were consistent (M = 0.47 s, Min = 0.47 s, Max = 0.5 s). In total, the decomposition with BiSect required 35.62 s and resulted in 103 fragments. The proposed approach was performed in ca. 3.52 min (1.69 min skeletonization and 1.83 min decomposition) and resulted in 487 fragments. Note that the runtime of MaxFat can be optimized by parallelizing the execution of the algorithm not only on the different polygons in one sample but also on the different skeleton branches.

### Practical results with LCM

The practical evaluation of both shape decomposition approaches consisted of the dissection of all computed fragments with LCM. Because one tissue sample cannot be dissected twice, the experiment was performed on empty microscopic slides. Therefore, it was not possible to compare the amount of successfully dissected tissue by measuring for example the protein content. The evaluation was restricted to visual assessment.

#### Classification of dissected fragments

The dissection of each fragment was observed and classified into the following categories. A fragment was labeled “*successful*” if it disappeared from the field of view after the laser pulse. In this case, we expect it to be successfully transferred into the collecting device. Unsuccessful fragments were further divided into three categories. The label “*torn*” describes fragments that tore during the dissection. Because they were only partially transferred, the collected area is not measurable. A fragment was labeled “*fallen*” in the following two cases. The fragment fell before the transferring process, which might be the case if all connections to the surrounding membrane were already severed before the laser pulse. The other case covers fragments that fell back onto the slide (in the field of view) after the laser pulse. One might only conjecture the reasons: As mentioned before, the size and shape of the fragments, the focus of the laser pulse as well as its position on the boundary of the fragment affect the transferring process and its success. The last category “*too small*” contains fragments of such a small size that the laser did not leave enough material to be collected.

**Fig. 14:**
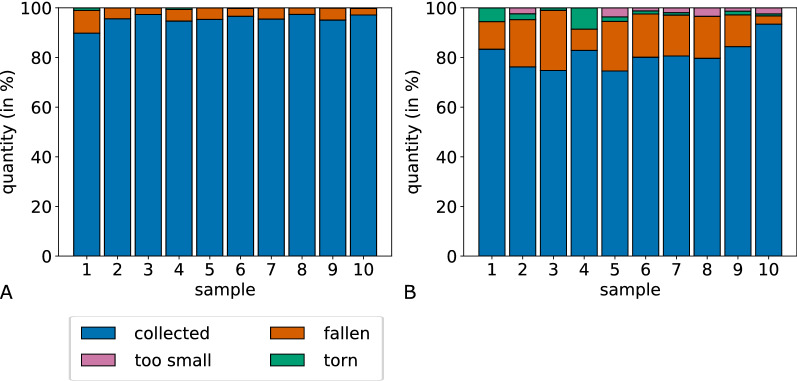
Distribution of labels assign during LCM. Comparison of the percentage of labels assigned to fragments during laser capture microdissection for MaxFat (**A**) and BiSect (**B**)

Figure 14 depicts the distribution of assigned labels based on the number of fragments in each category. In both decompositions, the majority of fragments was labeled as successful. The amount of successfully dissected fragments of MaxFat is consistently over 90% for all samples. The distribution for BiSect shows more variation between the samples. On average, 95.44% of the fragments of MaxFat were successful, 4.34% were labeled as fallen and merely 0.22% as torn. None of the fragments were too small. BiSect averages around 80.98% successful, 14.99% fallen, 2.39% torn and 1.64% too small fragments.

#### Area loss and success rates

**Fig. 15:**
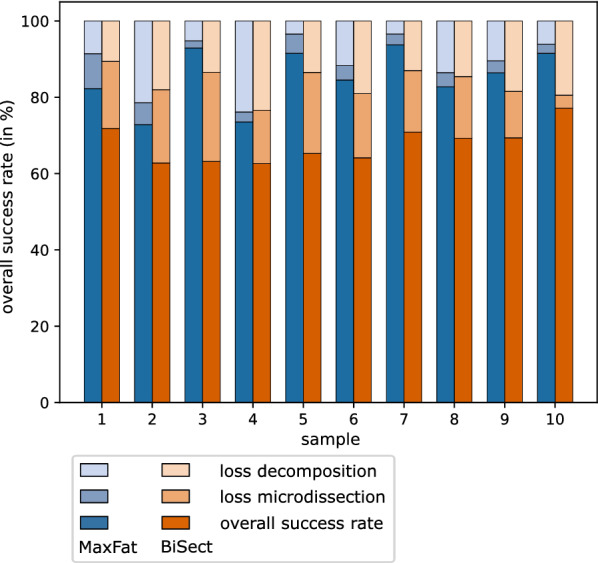
Comparison of success rates. Comparison of the amount of tissue loss and the overall success rates of MaxFat and BiSect. With 100% being the ROI area in the sample, the two lighter bar segments correspond to the percentage of tissue loss in the decomposition and the microdissection, respectively. The darkest segment represents the percentage of the original area that was successfully collected during the microdissection. This value represents the overall success rate

Tables 2, 3 show the success rates of MaxFat and BiSect with regard to tissue yield, the results are also visualized in Fig. 15. We distinguish two different success rates. The success rate of the microdissection (Table 2) represents the ratio of the area of the fragments that was successfully collected as computed by the LCM system. Over all ten samples, the values for MaxFat are higher than for BiSect. The largest difference can be observed for sample 3 with a value of 25.03%. Overall, the success rate for the microdissection averages at 95.46% for MaxFat and 80.99% for BiSect. Using these percentages, we calculated the overall success rate (Table 3) of both decomposition methods by combining the following factors: The ROI area contained in the samples (combined area of all components), the amount of area lost due to the decomposition algorithm and lastly the success rate of the microdissection. For example, the MaxFat decomposition resulted in 8.58% area loss for sample 1 (see Table 1). Thus, 91.42% of the original area was contained in the fragments for LCM. The microdissection showed a success rate of 90%, which results in an overall success rate of 82.28%. This means that 82.28% of the tissue contained in sample 1 could be collected using the MaxFat decomposition approach. For all ten samples, the overall success rate of MaxFat was at least 10% higher. In sample 3, the amount of lost tissue could potentially be decreased by 29.72% when using MaxFat rather than BiSect. On average, the tissue yield with the proposed decomposition approach is 17.55% higher.

The practical evaluation confirms our conjecture that the proposed decomposition method performs better in practice than the heuristic bisection approach. The amount of successfully dissected fragments is consistently higher with the MaxFat approach. With BiSect, this rate varies more noticeably and the algorithm was not able to filter out fragments that did not fulfill the lower area constraint. Besides the quantity of successfully dissected fragments, the tissue area that was collected with MaxFat was larger as well. Together with the smaller area loss in our decomposition, which we observed in the computational assessment, the proposed method proved to minimize the tissue loss considerably. When used on actual tissue samples, our decomposition method will increase the tissue yield and thus the amount of protein or DNA available for further analysis.

**Table 2:** Comparison of microdissection success rates for MaxFat and BiSect

sample	1	2	3	4	5	6	7	8	9	10
MaxFat	90.00	92.72	98.05	96.58	94.77	95.74	97.04	95.75	96.49	97.43
BiSect	80.39	76.61	73.02	81.79	81.79	79.19	81.47	81.09	85.10	95.80

The LCM success rate (in%) describes the amount of tissue area that was collected from the fragments by LCM

**Table 3:** Comparison of overall success rates for MaxFat and BiSect

sample	1	2	3	4	5	6	7	8	9	10
MaxFat	82.28	72.85	92.93	73.54	91.53	84.53	93.74	82.77	86.41	91.52
BiSect	71.85	62.79	63.21	62.64	70.78	64.13	70.88	69.26	69.38	77.16

This success rate (in%) represents the overall tissue yield from as sample by combining the LCM success rate with the area loss during the decomposition of components, i.e. how much area of the original ROI is contained in the computed fragments

## Conclusion

In this paper, we presented a skeleton-based decomposition method for simple polygons as a novel approach to decompose disease-specific regions in tissue samples while aiming to optimize the amount of tissue obtained by laser capture microdissection (LCM). The lack of previous benchmark methods and results is somewhat remarkable. It indicates that previous studies utilizing LCM relied on manual decomposition of the regions to be dissected, which is clearly impractical in clinical study settings involving dozens or hundreds of samples. As the first fully automated approach, we provide a conceptual contribution that may pave the way for making LCM feasible in large clinical studies. Our approach will also facilitate systematic assessment of optimal size and morphology criteria for LCM experiments, which would be difficult if not impossible to conduct based on manual shape decomposition.

Size and morphology of the fragments are assumed to be the key factors that influence the success of dissections using LCM. Our approach is designed to minimize tissue loss by utilizing a size constraint and optimizing the shapes towards fat or circular fragments. As we demonstrated, this translates into practice when comparing our approach to a recursive bisection method that is currently used and only applies a size constraint.

Our approach is tailored towards complex morphological structures that are commonly found in cancerous tissue and are usually the most challenging to dissect using LCM without major loss of tissue material. Not surprisingly, the algorithm does not perform as well when decomposing relatively large and fat shapes. However, such shapes do not occur frequently and the impact on the overall success seems to be minimal. These shapes can be easily decomposed using simple approaches, e.g. the bisection-based approach, without major tissue loss. Thus, we plan to improve our method by distinguishing these shapes and compute their decompositions separately.

The implementation of our approach relies on a (discrete) skeletonization of the underlying polygons. Specifically, we utilize the approach of Bai et al. [30], which uses a heuristic pruning approach. While other high-quality implementations of discrete skeletonization algorithms exist [38, 39], the approaches lack pruning strategies that are essential for our approach to produce practically relevant results. It is likely that recent improvements for skeletonization and pruning will further improve our results. For example, recent methods of Durix et al. [27, 28] promise to avoid short and other spurious branches, which contributed to area loss in our decomposition. More broadly, concepts for robust skeletonizations have been proposed based on the $\lambda$-medial axis [25, 40], which are built on solid theoretical grounds and thus may provide useful concepts for further improved shape decomposition approaches. Here, skeletonization is considered as an input to our decomposition method. While better and more robust skeletonization may further improve performance, a detailed investigation is beyond the scope of our present study.

The practical evaluation with LCM showed the advantage of using the proposed decomposition method. Over the entire sampling, our decompositions contained more successful fragments and we achieved a success rate of 95% in the dissection. In combination with the lower area loss during the decomposition, we were able to minimize the overall tissue loss for all samples and increased the tissue yield by 10–30%. Overall, our work contributes to further optimization and automation of LCM and thus promises to contribute to the further maturing of the technology and enhancing its suitability for systematic use in larger scale clinical studies.

## Data Availability

Source code and all shapes from our validation samples as well as computational results are available from https://github.com/Lahutar/Skeleton-based-Shape-Decomposition.git.

## Abbreviations

LCM: Laser capture microdissection
ROI: Region of interest
H[MYAMP: E-stain] Hematoxylin and eosin stain
FFPE: Formalin-fixed paraffin-embedded
M: Arithmetic mean
SD: Standard deviation
